# Dysplastic intestinal-type metaplasia of appendiceal endometriosis: a mimic of low grade appendiceal mucinous neoplasm

**DOI:** 10.1186/1746-1596-9-39

**Published:** 2014-02-21

**Authors:** Andrew Mitchell, Pierre Dubé, Lucas Sideris

**Affiliations:** 1 Department of Anatomic Pathology and Cytology, Maisonneuve-Rosemont Hospital, Montreal, Quebec, Canada; 2 Department of Surgery, Maisonneuve-Rosemont Hospital, Montreal, Quebec, Canada

**Keywords:** Appendix, Endometriosis, Intestinal, Metaplasia, Dysplasia

## Abstract

We report an example of dysplastic intestinal-type metaplasia involving endometriosis of the appendix in a 45 year old woman. One other example of this phenomenon has been reported. As it occurs within the muscular wall of the appendix, confusion with low grade appendiceal mucinous neoplasm (LAMN) may occur. Evidence supporting the metaplastic nature of the intestinal epithelium is offered. As the initial pathological diagnosis was of invasive cancer with perforation of the appendix treatment consisted of peritonectomy and hyperthermic intraperitoneal chemotherapy (HIPEC).

**Virtual slides:**

The virtual slides for this article can be found here: http://www.diagnosticpathology.diagnomx.eu/vs/1068246472111756.

## Introduction

Endometriosis of the appendix may be an incidental finding or the cause of appendicitis, intussusception or perforation [1]. Various types of metaplasia may involve the epithelial component of endometriosis [2] including intestinal-type. The latter has been described in two cases of appendiceal endometriosis to date, one associated with focal dysplasia [3,4]. We describe a case of endometriosis of the appendix with dysplastic intestinal-type epithelium presenting as a mucocele with acute appendicitis and perforation. The differential diagnosis is with low grade appendiceal mucinous neoplasm (LAMN). As the initial pathologic diagnosis was of “infiltrating low-grade adenocarcinoma colonizing endometriosis” the patient was treated with peritonectomy and hyperthermic intraperitoneal chemotherapy (HIPEC).

### Case details

A 45 year old woman underwent surgery at another hospital following a diagnosis of acute appendicitis. The appendix appeared enlarged and perforated. Multiple “peritoneal mucinous implants” were observed in the right pelvis. Following a pathologic diagnosis of “invasive low-grade carcinoma of the appendix colonizing appendicial endometriosis”, the patient was referred to our hospital for consideration of second look laparotomy and HIPEC. An extensive evaluation revealed no evidence of metastatic disease.

Without recourse to pathological review of the appendectomy specimen, second look laparotomy was performed exactly one year later. There was no macroscopic evidence of neoplasia, but mucin was seen localized to the right peritoneal surface. Free intrabdominal mucin was absent. Right hemicolectomy, resection of two segments of small intestine, omentectomy, bilateral ovariectomy, and peritoneal resections were carried out. HIPEC (Oxaliplatin 300 mg/m^2^) was administered. Pathologic examination showed foci of endometriosis on the ileal surface of the right hemicolectomy specimen, the left Fallopian tube and in one of the fragments of peritoneum. The mucin was acellular. Both ovaries had functional cysts.

The patient’s clinical course has been uneventful after eighteen months.

## Material and methods

The haematoxylin-phloxin-saffron (HPS) stained sections of the appendectomy specimen from the referring hospital were reviewed. In turn, 4- micron thick recuts of all the formalin-fixed, paraffin-embedded tissue blocks were stained with HPS in our laboratory. Unstained sections from all blocks were submitted for histochemical and immunohistochemical studies: periodic acid-Schiff stain with diastase (PAS-D) and without (PAS), and monoclonal antibodies directed against pancytokeratin AE/AE3, cytokeratin 7, cytokeratin 20, CD10, estrogen (ER) and progesterone (PR) receptors, and Ki-67 (MIB-1).

### Pathologic findings

**Figure 1:**
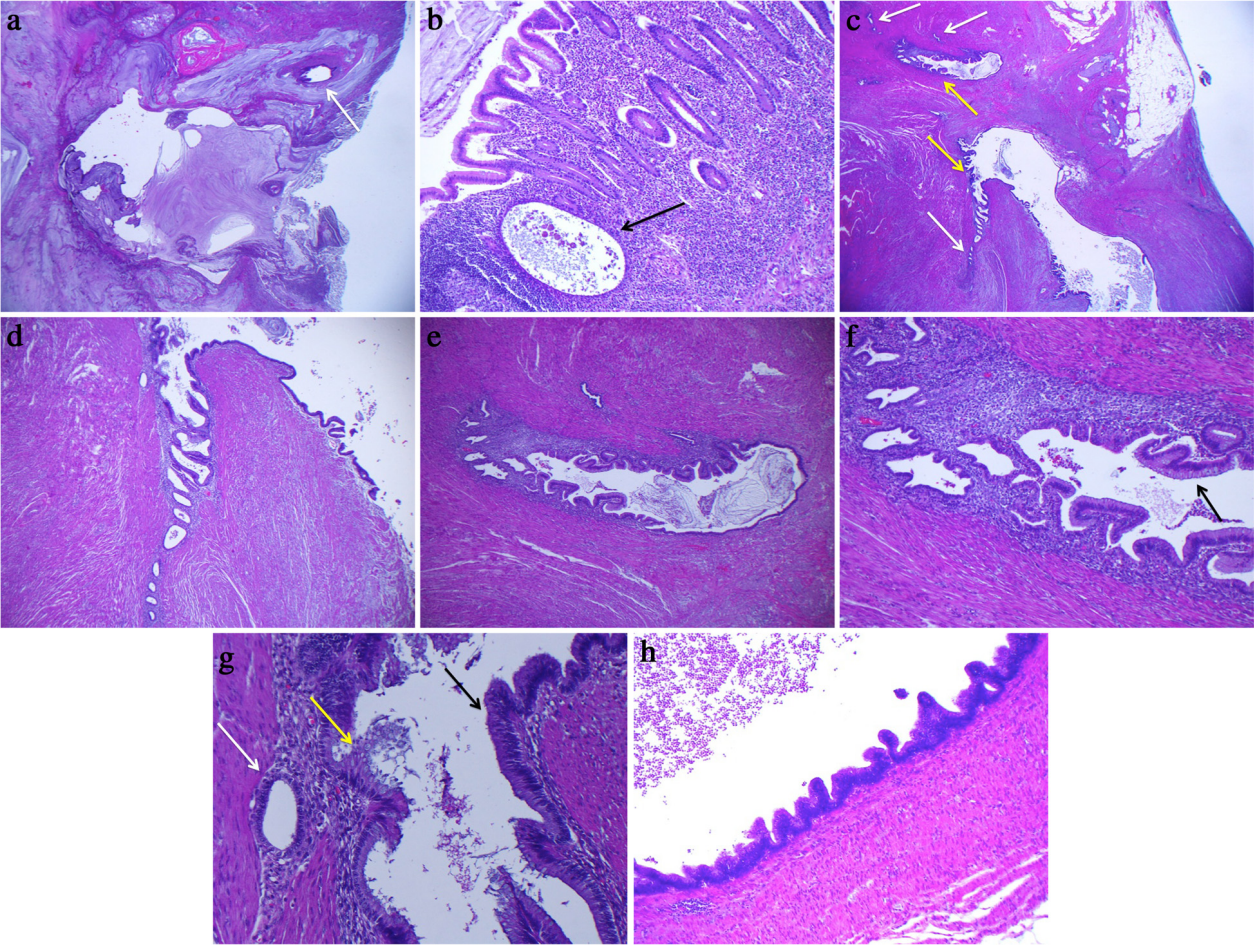
**Histologic findings. a)** Low power view of rupture site with free mucin and a dysplastic intestinal-type gland (arrow). **b)** Low power view of normal appendix epithelium with underlying endometriosis. **c)** Low power view of endometriosis of the appendix wall with transition to intestinal-type epithelium (arrows). **(d and e)** Further low power views of endometriosis of the appendix wall with transition to intestinal-type epithelium. **f)** Higher power view of 1e. Note intestinal-type epithelium (arrow). **g)** High power view of endometriosis, intestinal-type metaplasia, and intestinal-type metaplasia with low-grade atypia (arrows left to right). **h)** High power view of an area of tufting in low grade dysplasia.

The pathology report from the referring hospital described an appendix 6 cm in length and varying from 1 to 2,5 cm in diameter with presence of a mucocele and perforation. The wall varied from 0,5 to 1 cm in thickness with “brownish zones”. Microscopic examination showed acute appendicitis, acellular mucin dissecting the wall with extension to the serosa compatible with perforation (Figure 1a) and multiple foci of endometriosis involving the lamina propria and wall (Figures 1b and 1c). Within certain foci of endometriosis direct transition of endometriotic epithelium into intestinal-type mucinous epithelium was observed (Figures 1c and d). Whereas this epithelium rested in large part on stroma of endometriotic-type (Figure 1e) in other areas this stroma was absent and the epithelium was in direct contact with smooth muscle. Occasional Paneth cells and ciliated cells were identified. The metaplastic epithelium had foci of mild to moderate cytologic atypia, occasional mitoses and tufting (Figures 1f and g). Although endometriosis extended from the lamina propria (Figure 1b) to the subserosa, the luminal epithelium showed no continuity with endometriosis and no atypia beyond reactive changes due to inflammation. There was no evidence of an appendicial diverticulum. The mucin at the serosal surface was acellular.

**Figure 2:**
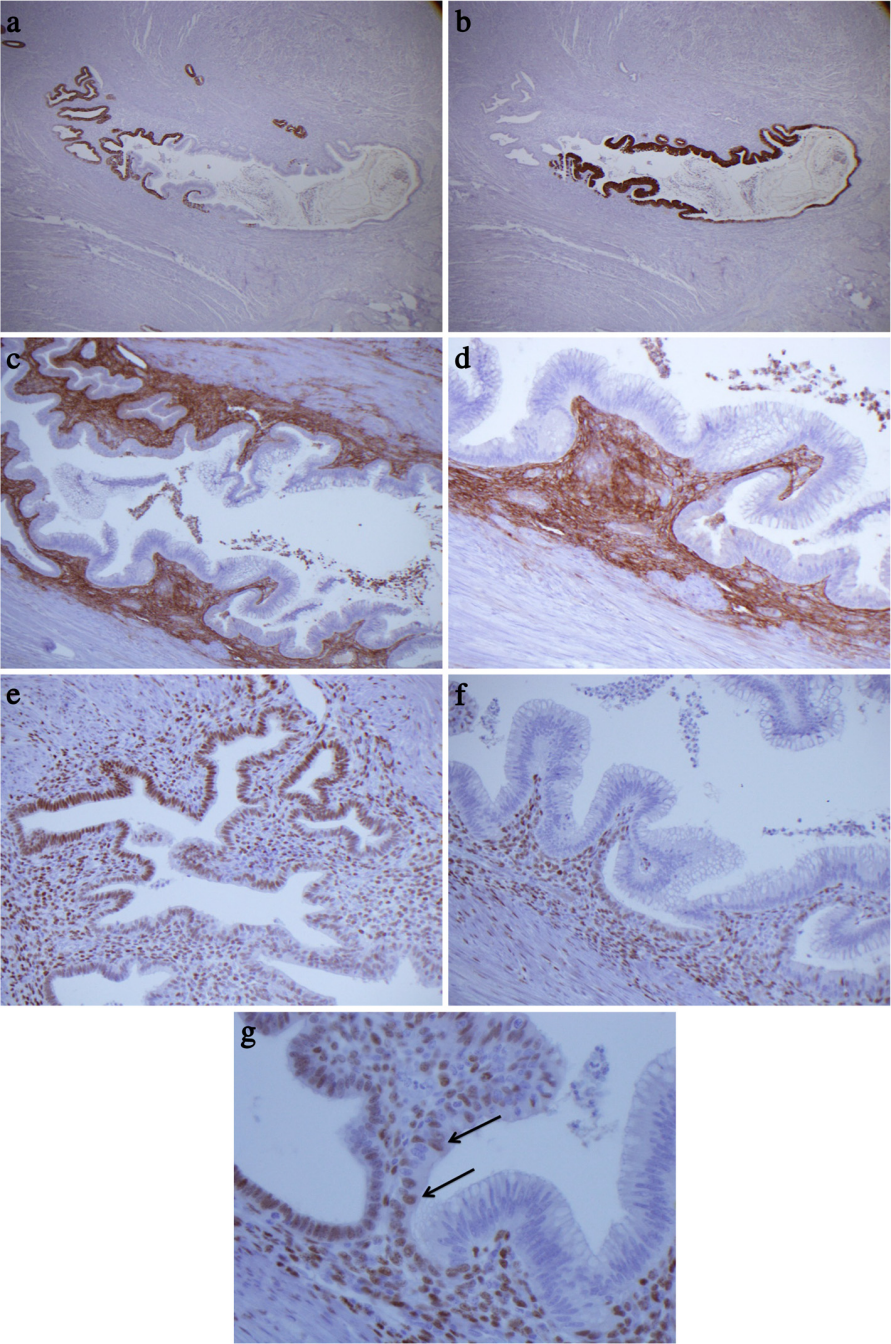
**Immunohistochemical findings. a)** Cytokeratin 7 positivity limited to endometriotic epithelium. **b)** Cytokeratin 20 positivity limited to intestinal-type epithelium. **c)** CD10 positivity of endometriotic stroma on which both endometriotic and intestinal-type epithelilia rest. **d)** CD10 positive endometriotic stroma surrounding intestinal-type glands. **(e and f)** ER positivity of endometriotic epithelium and stroma, but absence in metaplastic epithelium. **g)** Rare ER positive “transitional” cells with intestinal-type differentiation (arrows).

The foci of endometriosis showed a typical cytokeratin 7 positive/cytokeratine 20 negative epithelium (Figure 2a) with rare Ki-67 positive cells. The intestinal-type epithelium showed a cytokeratine 7 negative/cytokeratine 20 positive profile (Figure 2b) with Ki-67 positivity of at least 20%. The endometriotic stroma underlying both epithelia was CD10 positive (Figure 2c and d) and ER and PR positive (Figures 2e and 2f). The intestinal-type epithelium was ER negative (Figure 2f) although rare mucinous cells at the transition between endometriotic and mucinous epithelia were focally ER-positive (Figure 2g).

The findings were interpreted as acute appendicitis with perforation and background endometriosis, the latter with foci of metaplastic intestinal-type epithelium with occasional low grade dysplasia.

## Discussion

Endometriosis has been described in numerous extra-pelvic sites including the abdominal wall, pleura, pericardium, muscle, nerve [5] and bronchus [6]. Confusing clinical presentations may result. As one example, within the gynecologic tract co-existence of an ovarian leiomyoma with an endometriotic cyst resulted in appendicitis-like symptoms and urgent laparotomy [7].

Endometriosis may involve the gastrointestinal tract from the small intestine to the rectosigmoid colon. The latter is the most common site, followed, in order, by the proximal colon, small intestine, the appendix and cecum. Ileocolic intussusception due to cecal endometriosis has been documented [8].

Microscopic foci of endometriosis are encountered in appendices removed for appendicitis or those included in colectomy specimens, and are clinically silent. Conversely, endometriosis may present as acute or chronic appendicitis, with the latter occasionally causing a mucocele. Other presentations of appendicial endometriosis include a mass lesion, perforation during pregnancy (most commonly in the first two trimesters and when the endometriosis is transmural), and intussusception of the appendix [1].

Endometriosis can display metaplastic changes. In the ovary 12-68% of endometriosis lesions show metaplasia in the form of ciliated, eosinophilic, hobnail, squamous or mucinous change, with endocervical-type metaplasia encountered more commonly than intestinal-type [2]. Although in a review of 44 cases of endometriosis involving the intestinal tract no examples of intestinal-type metaplasia were found [1], a recent report documents intestinal epithelium “colonizing” endometriosis of the cecum of a 55 year-old woman who had previously undergone appendectomy [9].

One other case of dysplastic intestinal-type metaplasia involving appendicial endometriosis has been reported [3]. A 39 year-old woman with severe endometriosis underwent uterine myomectomy as well as appendectomy for an incidental 1.6 cm nodule of the distal appendix. Microscopic examination showed endometriosis extending from the serosa to the mucosa of the appendix. Foci of intestinal-type metaplasia, including Paneth cells, were found within the endometriosis with one area showing cytologic atypia consistant with dysplasia. Regarding histogenesis, the authors favored metaplasia of the endometriosis, as opposed to “colonization” by overlying luminal epithelium, citing the presence of ER-positive mucinous cells, the presence of ciliated mucinous cells (not seen in normal intestinal epithelium), absence of direct communication between endometriotic and native appendiceal glands, and the presence of “transitional epithelium”.

Similarly, we believe our case represents intestinal metaplasia rather than colonization, as: 1) no connection between the overlying native epithelium and the foci of endometriosis was found, 2) multiple foci of direct transition between endometriotic epithelium and intestinal-type epithelium were present, 3) ER-positive mucinous cells at sites of transition from endometriotic to mucinous epithelium were identified, and 4) the known potential of endometrium to undergo intestinal metaplasia [10,11].

The differential diagnosis in the present case is with low-grade appendiceal mucinous neoplasm (LAMN). LAMN is characterized by invasive intestinal-type epithelium with abundant mucin production, minimal architectural complexity and low-grade cytologic atypia [12]. Two features of the present case rule out LAMN: absence of invasive intestinal epithelium and absence of a primary lesion (adenoma or carcinoma in situ) of the appendicial epithelium. Furthermore, there is clinical support against LAMN as at second-look surgery one year following resection of the perforated appendix there was absence of both recurrent neoplasia and diffuse peritoneal mucin.

The decision to perform peritonectomy and HIPEC was based on an initial pathological diagnosis of invasive cancer. Given the uniqueness of our case it is impossible to know what further treatment, if any, would have been warranted had the correct diagnosis initially been made. We therefore suggest that in any appendectomy specimen with endometriosis and suspicion of neoplasia that the possibility of intestinal metaplasia be considered in the differential diagnosis.

## Consent

Written informed consent was obtained from the patient for the publication of this report and any accompanying images.

## Competing interests

The authors declare that they have no competing interests.

## Authors’ contributions

AM provided the pathologic diagnosis and drafted the manuscript. PD and LS provided clinical information, wrote the case details and contributed to the nonpathological aspects of the discussion. All authors read and approved the final manuscript.
